# Predictive Value of Pretherapeutic Maximum Standardized Uptake Value (Suv_max_) In Laryngeal and Hypopharyngeal Cancer

**DOI:** 10.1038/s41598-019-45462-y

**Published:** 2019-06-20

**Authors:** Jonas Werner, Martin W. Hüllner, Niels J. Rupp, Alexander M. Huber, Martina A. Broglie, Gerhard F. Huber, Grégoire B. Morand

**Affiliations:** 1 Department of Otorhinolaryngology - Head and Neck Surgery, University Hospital Zurich, Zurich, Switzerland; 2 Department of Nuclear Medicine, University Hospital Zurich, Zurich, Switzerland; 3 Department of Pathology and Molecular Pathology, University Hospital Zurich, Zurich, Switzerland; 4 Department of Otorhinolaryngology – Head and Neck Surgery, Kantonsspital St. Gallen, St. Gallen, Switzerland; 5 University of Zurich, Zurich, Switzerland

**Keywords:** Head and neck cancer, Surgical oncology

## Abstract

The aim of the study was to evaluate whether pretherapeutic metabolic tumor parameters from 18-fluorodeoxyglucose positron emission tomography (FDG-PET) imaging could predict larynx preservation in laryngeal and hypopharyngeal cancer patients prior to primary chemoradiation. Tumor metabolic parameters [maximum standardized uptake value (SUV_max_), metabolic tumor volume (MTV), and total lesion glycolysis (TLG)] were retrospectively assessed in a consecutive cohort of laryngeal and hypopharyngeal cancer patients undergoing primary (chemo-)radiation. Main outcome measures were larynx preservation and survival. The study included 97 patients with a median follow-up of 32 months (IQR 20–54.5). For hypopharyngeal cancer, multivariable analysis showed that patients with a primary tumor’s SUV_max_ > 9.5 entailed a higher risk of undergoing salvage pharyngolaryngectomy after chemoradiation (HR = 8.64, 95% CI = 1.1–67.3, *P* = 0.040). In laryngeal cancer, SUV_max_ did not predict the need for salvage laryngectomy. The only predictor for larynx preservation in laryngeal cancer patients was T-classification at initial diagnosis (HR = 6.67, 95% CI = 0.82–53.9, *P* = 0.039). In conclusion, SUV_max_ of primary tumor could be used as a predictor of larynx preservation prior to primary chemoradiation in hypopharyngeal cancer patients. This information may be important for patient counseling, as high SUV_max_ was correlated with reduced probability of larynx preservation. However, in laryngeal cancer patients, SUV_max_ does not seem to be predictive of outcome.

## Introduction

In advanced stage III or IV laryngeal and hypopharyngeal squamous cell carcinoma, organ preservation strategy involves primary chemoradiation as first-line therapy, reserving surgery such as laryngectomy or pharyngolaryngectomy for the salvage setting^1,2^. Primary upfront laryngectomy or pharyngolaryngectomy followed by adjuvant radiotherapy with or without concomitant chemotherapy, as needed, may be preferred in patients with surgically removable advanced stage carcinomas, as primary chemoradiation with salvage surgery may negatively impact quality of life, functionality and survival^3^. Therefore, it is crucial to adequately select patients for either of the two strategies. Tumor response to induction chemotherapy has been used in seminal studies as a selection tool^2^. However, subsequent studies revealed the superiority of concurrent chemoradiation compared to induction chemotherapy followed by radiotherapy for laryngeal preservation, establishing a new standard of care^4^. Although a few clinical factors, such as gross cartilage infiltration, large tumor volume, and extensive nodal disease are already recognized as unfavorable factors for organ preservation strategy, it would be of interest to provide further tools for clinicians to base their therapeutic decision on^5^.

18-fluorodeoxyglucose positron emission tomography (FDG-PET) with computed tomography (CT) or magnetic resonance (MR) imaging has become a broadly accepted imaging tool in routine clinical oncology^6^. Adding FDG-PET to the staging process resulted in higher staging accuracy with improved nodal classification^7,8^, superior detection of regional or distant metastases, and second primary cancers^9,10^. In addition, metabolic tumor parameters derived from FDG-PET have been suggested to serve as prognostic markers for the response to chemoradiation^11,12^. Such metabolic parameters include maximum standardized uptake value (SUV_max_), metabolic tumor volume (MTV), and total lesion glycolysis (TLG)^13,14^.

Tumors with a higher uptake of FDG have a more active tumor metabolism, which negatively correlates with tumor oxygenation through the Warburg effect^15,16^. Poor tumor oxygenation or tumor hypoxia is moreover associated with resistance to chemoradiation^17,18^. Consequently, we hypothesized that FDG-PET derived tumor metabolism markers, such as SUV_max_, MTV, and TLG, can be used as predictors of response to chemoradiation. The aim of this study was therefore to examine whether different pretherapeutic FDG-PET parameters can predict organ preservation in laryngeal and hypopharyngeal cancer undergoing primary chemoradiation.

## Materials and Methods

### Study population

After Ethics Review Board approval by the *Kantonale Ethikkomission Zürich* (protocol number 2016–01799), all patients treated for laryngeal or hypopharyngeal squamous cell carcinoma between June 1^st^, 2007, and June 1^st^, 2017, at the Department of Otorhinolaryngology – Head and Neck Surgery of the Zurich University Hospital, Switzerland, were retrospectively assessed. All research was performed in accordance with relevant guidelines and regulations and informed consent of all enrolled patients was obtained. Inclusion criteria were available pre- and posttherapeutic FDG-PET/CT or FDG-PET/MR images and treatment with curative intent. Patients treated with induction chemotherapy, patients that did not complete a course of radiotherapy of at least 60 Gray locally, and patients undergoing primary surgical treatment were excluded.

All patients were staged according to the *Union Internationale Contre le Cancer* (UICC), TNM staging for head and neck cancer, 7th edition, 2010^19^. After full medical history, physical examination, triple endoscopy with biopsy, and imaging with FDG-PET, all patients were presented and discussed at the local interdisciplinary tumor board. Posttherapeutic FDG-PET/CT or -/MR images were obtained three months after completion of chemoradiation.

Detailed data on age, gender, tumor subsite, and risk factors including smoking, drinking habits, and human papilloma virus (HPV) status were obtained. Immunohistochemical expression of p16 or polymerase chain reaction (PCR) for HPV were used to evaluate HPV status of tumor biopsy samples. Local and regional radiation dose, type and number of cycles of concomitant chemotherapy, time to salvage pharyngolaryngectomy and/or neck dissection, pathological tumor stage, number of nodes dissected, number of positive nodes, and follow-up time were assessed.

The study cohort was then divided into two groups according to tumor site, analyzing laryngeal and hypopharyngeal cancer separately. The primary outcome measure of the study was pharyngo-/laryngectomy-free survival. Secondary outcome measures included local and regional recurrence-free survival, distant metastasis-free survival, disease-specific survival, and overall survival.

### FDG-PET/CT or -/MR image acquisition

Patients were injected with a standardized dose of 3.5 MBq of 18-fluorodeoxyglucose (FDG) per kilogram body weight after fasting for at least four hours. All patients had a blood glucose level below 12 mmol/l before imaging. The patients were instructed to remain in lying or recumbent position and silent for 50–60 minutes to minimize muscular FDG uptake in the period between FDG injection and image acquisition. Patients were also kept warm prior to tracer injection and throughout the uptake period to diminish FDG accumulation in brown adipose tissue. All patients received either iodinated or gadolinium-based contrast medium. An integrated Discovery VCT PET/CT system (GE Healthcare, Waukesha, WI), a Discovery PET/CT 690 (GE Healthcare), or a hybrid PET/MRI system (Signa PET/MR, GE Healthcare) was used for image acquisition.

### Tumor FDG metabolism

Selected parameters of tumor FDG metabolism were obtained under supervision of a board-certified nuclear physician and radiologist and comprised pre- und posttherapeutic SUV_max_, TLG, and MTV of the primary tumor. Moreover, in patients with clinically positive nodal status, SUV_max_ of the most active metastatic lymph node was measured. SUV_max_ was calculated automatically using a standard formula [maximum activity in region of interest ÷ (injected dose × body weight)]. MTV was defined as the sum of the volume of voxels with an SUV exceeding a threshold of 42% of the SUV_max_. TLG was defined mathematically as MTV × SUV_mean_. Correct analysis of FDG uptake was ensured through side-by-side reading of the corresponding CT or MR images of the tumor in the axial, coronal, and sagittal plane. Borders of regions of interest (ROI) were set by manual adjustment to exclude adjacent physiologic FDG-avid structures. A written report by a dually board-certified nuclear medicine physician/radiologist was available for pre- and posttherapeutic FDG-PET/CT or -/MR images.

### Statistical analysis

For continuous variables, median, interquartile range (IQR), or standard deviation (SD) are given. To compare distribution among samples, the non-parametric Mann Whitney U test was used for two samples. Binary variables were associated in contingency tables using the two-tailed chi-squared test. Main outcome measures of the study were calculated using a multivariable Cox regression model. Results are expressed in hazard ratio (HR) with the 95% confidence interval provided (95% CI). Survival curves were built according to Kaplan-Meier and the log-rank test was used to compare factors. Receiver operating characteristic (ROC) curves were used to determine in which study group pretherapeutic SUV_max_ was a potential predictor of laryngectomy and to select the best cutoff value for SUV_max_ to predict high risk of laryngectomy. A *P*-value lower than 0.05 was considered to indicate statistical significance. Statistical analyses were performed using SPSS® 23.0.0.0 software (IBM®, Armonk, NY, USA).

### Meeting presentation

This work was presented at the 2018 Swiss Society for Oto-rhino-laryngology Head and Neck Surgery, Spring Meeting, June 21^st^, 2018, Basel, Switzerland.

## Results

### Patient and tumor characteristics

A total of 97 patients with advanced stage III or IV laryngeal or hypopharyngeal cancer were included in this study (Table 1). The median age at diagnosis was 64 years (IQR 56–70). As expected, there was a clear male predominance with 85 (87.6%) male and 12 (12.4%) female patients. Forty patients (41.2%) had squamous cell carcinomas of the larynx and 57 patients (58.8%) of the hypopharynx. Most patients (58.8%) had cT3-cT4 tumors in comparison to 41.2% of patients with cT1-cT2 tumors, including glottic carcinomas. Clinical nodal status was positive in 61 patients (62.9%), of which 40 (41.3%) were staged with cN1-cN2b and 21 (21.6%) with cN2c-cN3 categories.

**Table 1:** Patient Demographics and Clinical Characteristics.

Variable		All patients No. of patients = 97	Hypopharynx (H) No. of patients = 57	Larynx (L) No. of patients = 40	*P* value^a^ H vs. L
***Age***					0.184
Years	Median (Q25-75)	64 (56–70.5)	64 (58–71)	62.5 (54–70)	
***Gender***					0.199
Male	n (%)	85 (87.6%)	52 (91.2%)	33 (82.5%)	
Female	n (%)	12 (12.4%)	5 (8.8%)	7 (17.5%)	
***Smoking***	Present (%)	94 (96.9%)	55 (96.5%)	39 (97.5%)	0.778
	Absent (%)	3 (3.1%)	2 (3.5%)	1 (2.5%)	
Pack years	Median (Q25-75)	40 (30–60)	40 (20–60)	50 (40–80)	0.014*
***Alcohol abuse***	Present (%)	49 (50.5%)	32 (56.1%)	17 (42.5%)	0.186
	Absent (%)	48 (49.5%)	25 (43.9%)	23 (57.5%)	
***p16/ HPV PCR***	Positive	8 (8.3%)	6 (10.5%)	2 (5%)	0.681
	Negative	37 (38.1%)	25 (43.9%)	12 (30%)	
	n/a	52 (53.6%)	26 (45.6%)	26 (65%)	
***PET imaging***					
SUV_max_ primary tumor	Median (Q25-75)	10.4 (7.4–15.7)	12.3 (9.2–15.3)	8.8 (6.7–16.6)	0.062
TLG primary tumor	Median (Q25-75)	34696 (18160–75166)	42926 (21467–77672)	29060 (15555–55644)	0.054
MTV primary tumor (cm^3^)	Median (Q25-75)	6.1 (3.6–8.7)	6.1 (3.6–9.7)	5.9 (3.5–8.2)	0.393
SUV_max_ nodal (cN+)	Median (Q25-75)	9.7 (7.3–11.8)	10.8 (7.5–12.3)	8 (6.8–10)	0.041*
***Pharyngo-/Laryngectomy***	Yes (%)	23 (23.7%)	14 (24.6%)	9 (22.5%)	0.814
	No (%)	74 (76.3%)	43 (75.4%)	31 (77.5%)	
***T-classification***					0.99
T1-T2	n (%)	40 (41.2%)	24 (42.1%)	16 (40%)	
T3-T4	n (%)	57 (58.8%)	33 (57.9%)	24 (60%)	
***N-classification***					<0.001*
N0	n (%)	36 (37.1%)	10 (17.5%)	26 (65%)	
N1	n (%)	11 (11.3%)	8 (14%)	3 (7.5%)	
N2a-b	n (%)	29 (30%)	23 (40.4%)	6 (15%)	
N2c-N3	n (%)	21 (21.6%)	16 (28.1%)	5 (12.5%)	

^a^Mann-Whitney U Test for continuous variables, 2-sided Pearson Chi-Squared Test for categorical variables.

SUV_max_: maximum standard uptake value. TLG: total lesion glycolysis. MTV: metabolic tumor volume.

*P* value for null hypothesis; *statistically significant.

The median pretherapeutic SUV_max_ was 10.4 (IQR 7.4–15.7) for the whole cohort, while the median TLG was 34696 (IQR 18160–75166), and median MTV was 6.1 cm^3^ (IQR 3.6–8.7 cm^3^). Median follow-up time for all patients was 32 months (IQR 20–54.5).

### Treatment characteristics

Patients received either intensity-modulated radiotherapy (IMRT) or volumetric modulated arc therapy (VMAT) with a mean total dose of 70 Gray locally (SD 1.65) and 54 Gray (SD 0.83) regionally. Seventy-four patients (76.3%) received concomitant chemotherapy. 63.5% of chemotherapies were based on cisplatin with a median of five cycles (SD 0.97), while 17.6% consisted of cetuximab with a median of four cycles (SD 1.81). Fourteen patients (18.9%) received both cisplatin and cetuximab, either based on the study protocol of a clinical trial^20^ or because the therapy was changed to cetuximab due to adverse effects of cisplatin.

Twenty-three patients (23.7%) underwent salvage surgery. Of those, 60.9% had hypopharyngeal cancer and underwent pharyngolaryngectomy and 39.1% had laryngeal cancer with salvage laryngectomy. Median time to pharyngo-/laryngectomy was 13.5 months (IQR 8.8–16.3). Frozen sections were used intraoperatively to assure free margins of the surgical resection specimen and all patients had negative margins upon final pathology (R0).

Neck dissection was performed either on its own or combined with pharyngo-/laryngectomy on 35 patients (36.1%) after a median time of 7 months (IQR 6–14). Eight of these patients (22.8%) had laryngeal cancer in comparison to 27 hypopharyngeal carcinomas (77.2%). Twenty of 35 patients (57.1%) had positive nodal disease (ypN+) with a mean of 2.3 positive lymph nodes (SD 2.59).

### Primary Outcome Analysis: Organ Preservation

**Figure 1:**
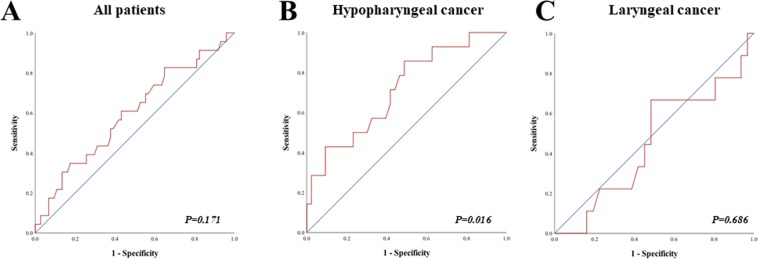
Receiver operating characteristic (ROC) curve analysis of laryngectomy prediction according to pretherapeutic SUV_max_ of primary tumor. (**A**) ROC curve for all patients showing lack of significant correlation (area under the curve (AUC) = 0.595 (95% CI = 0.460–0.730, P = 0.171). (**B**) ROC in hypopharyngeal cancer patients only. The area under the ROC curve was 0.715 (95% CI = 0.562–0.868, P = 0.016) and 9.5 was determined as best potential cutoff value for comparison. The sensitivity and specificity for SUV_max_ = 9.5 were 92.9% and 37.2%, respectively. (**C**) ROC curve in laryngeal cancer patients showing lack of significant correlation (AUC = 0.455 (95% CI = 0.241–0.699, P = 0.686).

Various cutoff values for FDG uptake parameters were tested for the different study cohorts. Using receiver operating characteristic (ROC) curves, it was determined that pretherapeutic SUV_max_ was predictive of laryngectomy for hypopharyngeal cancer patients but not for laryngeal cancer patients (Fig. 1, Panel A–C). For hypopharyngeal cancer patients, the best potential cutoff value for pretherapeutic SUV_max_ was determined to be 9.5 (Fig. 1, Panel B, sensitivity 92.9%, specificity 37.2%, *P* = 0.016). Factors possibly predicting organ preservation were analyzed separately between hypopharyngeal (Table 2) and laryngeal cancer patients (Table 3), using a univariable and multivariable Cox regression model.

**Table 2:** Cox regression analysis for pharyngolaryngectomy-free survival for hypopharyngeal cancer patients (No. of patients = 57).

Variable	Univariable analysis			Multivariable analysis		
	HR	95% CI	*P* value	HR	95% CI	*P* value
*Gender* Male vs. Female	1.37	0.18–10.5	0.760			
*Age* ≥ 70 vs. <70 years	0.58	0.19–1.72	0.342			
*T-classification* T3 + T4 vs. T1 + T2	3.77	1.05–13.6	0.028*	3.49	0.96–12.6	0.057
*N-classification* N2 + N3 vs. N0 + N1	2.69	0.60–12.1	0.194			
*Alcohol abuse* Present vs. absent	0.43	0.14–1.30	0.139			
*Smoking* Present vs. absent	0.56	0.73–4.31	0.577			
*SUV*_*max*_ *primary tumor* ≥ 9.5 vs. <9.5	8.89	1.12–70.1	0.013*	8.64	1.10–67.3	0.040*
*TLG primary tumor* ≥ 20k vs. <20k	5.53	0.71–42.9	0.102			
*MTV primary tumor* ≥ 6 vs. <6 cm^3^	1.71	0.59–4.99	0.318			

HR: hazard ratio. 95% CI: 95% confidence interval. SUV_max_: maximum standard uptake value. TLG: total lesion glycolysis. MTV: metabolic tumor volume.

*P* value for null hypothesis; *statistically significant.

**Table 3:** Univariable analysis for laryngectomy-free survival for laryngeal cancer patients (No. of patients = 40).

Variable	Univariable analysis		
	HR	95% CI	*P* value
*Gender* Male vs. Female	30.9	0.04–219	0.305
*Age* ≥ 70 vs. <70 years	29.1	0.28–296	0.340
*T-classification* T3 + T4 vs. T1 + T2	6.67	1.12–53.9	0.039*
*N-classification* N2 + N3 vs. N0 + N1	0.49	0.06–3.98	0.508
*Alcohol abuse* Present vs. absent	1.09	0.29–4.07	0.895
*Smoking* Present vs. absent	20.45	0.01–734	0.876
*SUV*_*max*_ *primary tumor* ≥ 9.5 vs. <9.5	0.56	0.13–2.26	0.407
*TLG primary tumor* ≥ 20 K vs. <20 K	0.79	0.19–3.25	0.753
*MTV primary tumor* ≥ 6 vs. <6 cm^3^	0.73	0.19–2.73	0.641

HR: hazard ratio. 95% CI: 95% confidence interval. SUVmax: maximum standard uptake value. TLG: total lesion glycolysis. MTV: metabolic tumor volume.

*P* value for null hypothesis; *statistically significant.

**Figure 2:**
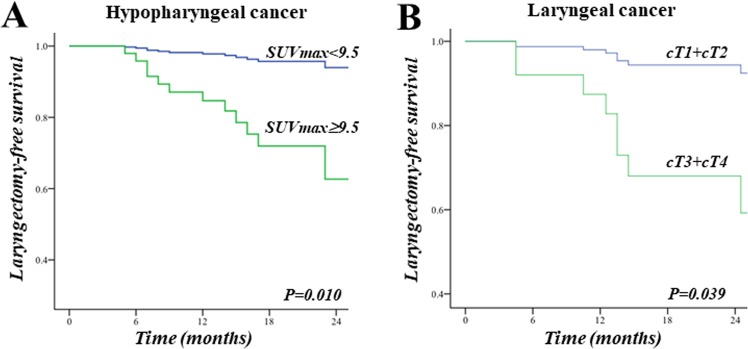
Kaplan-Meier curves showing laryngectomy-free survival. (**A**) High SUV_max_ predicted poorer laryngectomy-free survival in hypopharyngeal cancer patients (Log rank test, P = 0.010). (**B**) In laryngeal cancer patients, laryngectomy-free survival was predicted by T-category before chemoradiation.

In hypopharyngeal cancer, univariable analysis showed that pretherapeutic SUV_max_ ≥ 9.5 and T3/4 classification were predictors for salvage pharyngolaryngectomy. In multivariable analysis, the only independent predictor of salvage laryngopharyngectomy was a pretherapeutic SUV_max_ of the primary tumor ≥ 9.5 (HR = 8.64, 95% CI = 1.1–67.3, *P* = 0.040). Comparative Kaplan-Meier survival analysis showed a worse laryngectomy-free survival in patients with pretherapeutic SUV_max_ ≥ 9.5 (Fig. 2A, Log rank test, *P* = 0.010).

**Figure 3:**
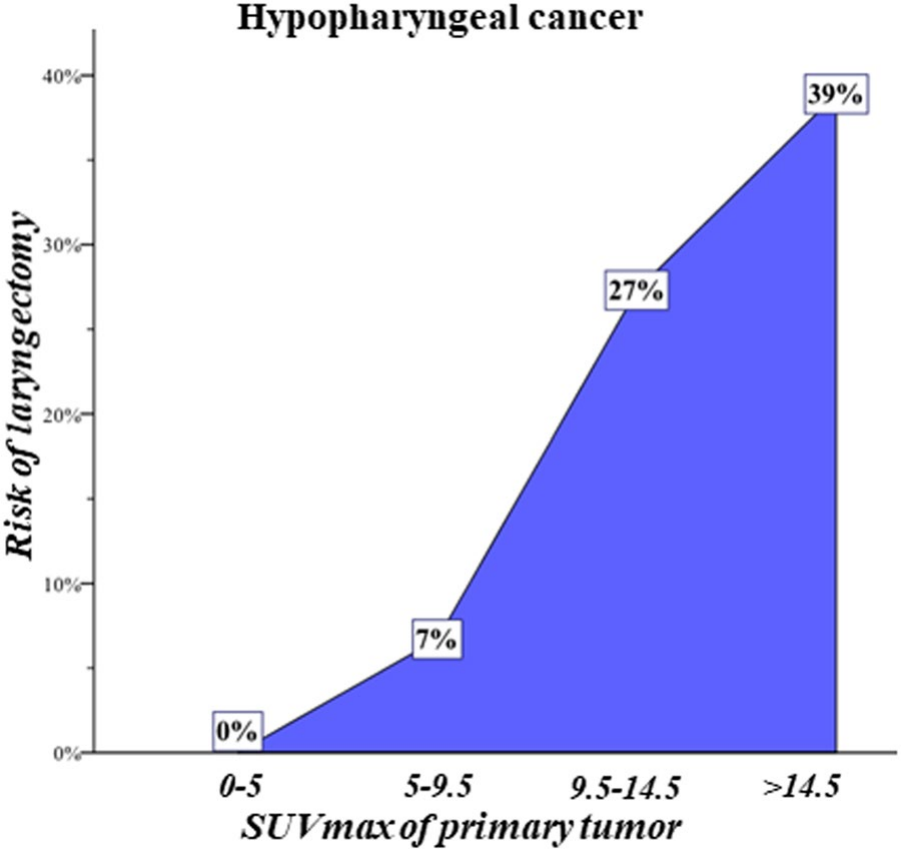
Frequency curve showing risk of laryngectomy according to SUV_max_ of primary tumor arranged in ordinal fashion. For patients with low SUV_max_ (<9.5), risk of laryngectomy was low. The risk of laryngectomy increased with higher SUV_max_ in an almost linear manner.

The risk of salvage pharyngolaryngectomy according to pretherapeutic SUV_max_ of the primary tumor was also evaluated in ordinal fashion. For patients with pretherapeutic SUV_max_ < 9.5, risk of laryngectomy was low. The risk of laryngectomy increased with a higher pretherapeutic SUV_max_ in an almost linear manner, as depicted in Fig. 3.

In laryngeal cancer, the only predictor of laryngeal preservation was T-classification (HR = 6.67, CI = 0.82–53.9, *P* = 0.039) in univariable analysis. Gender, age, N-classification, smoking, alcohol abuse, and metabolic tumor parameters (pretherapeutic SUV_max_, MTV, TLG) were not predictors of organ preservation (Table 3, each P > 0.05).

As expected, a low posttherapeutic SUVmax (<3.0) was also predictive of organ preservation in laryngeal and hypopharyngeal cancer (Log-rank test P = 0.001, not shown)

### Secondary outcome analysis: survival

**Figure 4:**
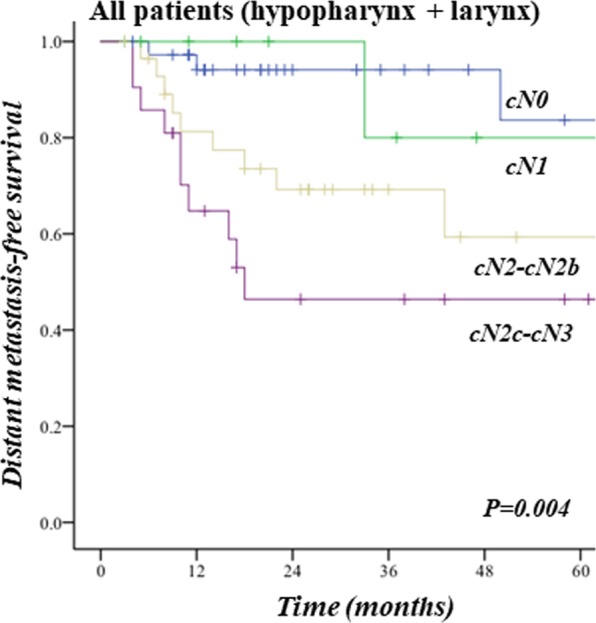
Kaplan-Meier analysis showing distant metastasis-free survival in hypopharyngeal and laryngeal cancer patients according to nodal status (Log rank test, P = 0.004).

The cumulative distant metastasis-free survival at 60 months was 69%. A majority of patients (58.3%) presented with pulmonary metastases. The only factor significantly predictive of distant metastases was nodal stage at diagnosis (Fig. 4, log-rank test, *P* = 0.004). The cumulative disease-specific survival at 60 months was 61%, with most events occurring within the first three years of follow-up. The cumulative overall survival at 60 months was 49%.

Pretherapeutic SUV_max_ of the primary tumor was not predictive of regional recurrence-free survival, distant metastasis-free survival, disease-specific survival, and/or overall survival (not shown, log-rank test, each P > 0.05).

Posttherapeutic SUVmax of the primary tumor was predictive of distant metastasis-free survival, disease-specific survival (log-rank test, P = 0.046 and P = 0.021, respectively) but not of regional recurrence-free and overall survival (log-rank test, each P > 0.05).

## Discussion

This study evaluates whether pretherapeutic metabolic parameters of laryngeal and hypopharyngeal cancer patients can predict tumor response to chemoradiation. Pretherapeutic SUV_max_ of the primary tumor was a predictor of laryngeal preservation before chemoradiation in hypopharyngeal cancer but did not seem to be predictive of organ preservation in laryngeal cancer.

The primary goal of our study was to identify pretherapeutically available markers to assist in clinical decision making, choosing between primary chemoradiation and primary surgery followed by adjuvant radiotherapy, as needed.

We also analyzed posttherapeutic metabolic parameters and showed, in accordance with previously published literature^21,22^, that a high posttherapeutic SUV_max_ is indicative of poor metabolic response to chemoradiation and predictive of the need for salvage laryngectomy and worse distant metastasis-free and disease-specific survival.

**Figure 5:**
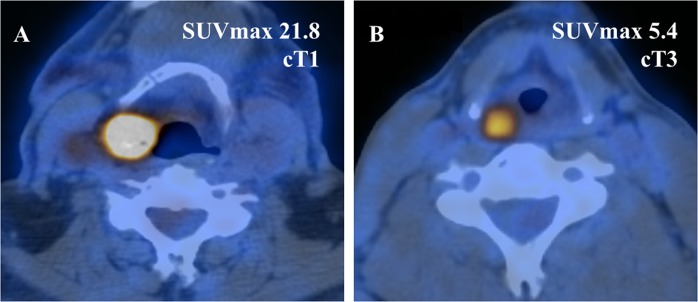
Representative axial fusion PET/CT images demonstrating two hypopharyngeal cancer patients. (**A**) This patient presented with a high SUV_max_ at diagnosis and underwent salvage laryngopharyngectomy. (**B**) This patient’s tumor exhibits a low SUV_max_ and responded completely to chemoradiation.

**Figure 6:**
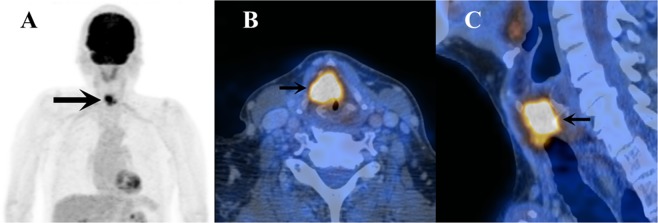
Example of a cT3 glottic carcinoma of the larynx in a 78-year-old patient. The tumor displays a SUV_max_ of 10.4, an MTV of 7 cm³, and a TLG of 45’830. (**A**) Frontal view of PET image. (**B**) Axial view of fused PET/CT image. (**C**) Sagittal fusion PET/CT image of the tumor.

Tumor hypoxia is known to adversely affect tumor response to radiotherapy^17^, attributed to a decrease of radiation-induced DNA damage under reduced oxygen conditions^23^. Hypoxic microenvironment within tumors alters cellular metabolism and triggers a myriad of molecular responses including upregulation of hypoxia inducible factors (HIFs)^24^. HIFs in turn promote transcription activation with upregulation of *SNAIL* and *TWIST* signaling pathways, resulting in activation of epithelial to mesenchymal transition (EMT)^25,26^. Hypoxia and HIFs also induce expression of *GLUT1* to provide the cell with sufficient energy through increased glucose uptake, including also FDG^27^. Our hypothesis was therefore that FDG-PET metabolic parameters, such as SUV_max_, could be used as surrogate markers of tumor hypoxia and poor prognosis. This hypothesis could be confirmed in hypopharyngeal (as Fig. 5 shows in an exemplary way) but not in laryngeal cancer. For the latter, T-classification at diagnosis was predictive of laryngeal preservation. Although we do not know the reason for this discrepancy, one factor might be the unassessed confounding effect of cartilage infiltration, which is a known predictor of poor response to chemoradiation, that may, however, be insufficiently addressed by the TNM classification^19^. Of importance, T-classification should not be underscored in hypopharyngeal cancer patients, as T3/T4 tumors tended towards poorer laryngectomy-free survival. The two cancer entities also differ in their tendency towards nodal metastasis^28^. Hypopharyngeal carcinomas are generally considered to develop nodal metastases earlier and more frequently compared to laryngeal cancer^29,30^. Comparing advanced stage hypopharyngeal and laryngeal carcinomas, patients with hypopharyngeal cancer are hence more likely to present with advanced nodal disease while those with cancer of the larynx are more likely to have a larger primary tumor to be categorized as advanced stage (Fig. 6 shows an example of a stage III (cT3 cN0 cM0) laryngeal cancer). This may feature another confounding factor in the analysis of tumor response to radiotherapy, although we did adjust for it in our multivariable analysis.

To the best of our knowledge, our study is the first to show that pretherapeutic SUV_max_ could help clinicians in decision-making to adequately select hypopharyngeal cancer patients suitable for organ preservation therapy. Nevertheless, previous studies have already suggested the potential of FDG-PET parameters in the prediction of organ preservation. Park *et al*.^14^ retrospectively analyzed the prognostic value of hypopharyngeal and laryngeal cancer metabolism in FDG-PET imaging before treatment. They identified MTV as an independent prognostic factor for both locoregional control (HR = 3.141, 95% CI = 1.175–8.399, *P* = 0.018) and overall survival (HR = 3.758, 95% CI = 1.415–9.982, *P* = 0.008). However, they did not separate upfront surgery with adjuvant radio(chemo)therapy from primary chemoradiation with salvage surgery, when needed. In their analysis of DeLOS-II trial, a German multicenter randomized phase II trial investigating functional organ preservation in patients with laryngeal and hypopharyngeal cancer receiving induction chemotherapy with or without cetuximab followed by radiotherapy, Wichmann *et al*.^5^ proposed a score facilitating decision-making between laryngectomy and organ preservation strategy based on the tumor’s early response to induction chemotherapy. Their score, identifying patients benefitting from larynx preservation strategies and those unsuitable for it, included the number of positive nodes, residual tumor volume, and a ratio of residual SUV_max_ to SUV_mean_ above 1.51 after induction chemotherapy^5^.

In our study, SUV_max_ was the one FDG-PET parameter with the best predictive value regarding organ preservation. Although other studies have recently emphasized on volumetric FDG-PET parameters such as MTV or TLG^13,31^, SUV_max_ has the advantage to be a standardized and easily applicable measure with the highest availability^32^. In clinical practice, it is better reproducible and less subject to variation due to different definitions of the region of interest or due to spill-over of adjacent FDG-avid structures^33^.

Based on our statistical analysis, we propose an SUV_max_ cutoff of 9.5 to distinguish between hypopharyngeal tumors with high and low risk of salvage pharyngolaryngectomy. In a previous study on oral cancer, our group already reported an SUV_max_ cutoff of 9.5^34^. Other studies assessing the association between SUV_max_ and survival reported cutoff values between 8.0 and 9.0 to identify head and neck carcinomas at risk of shorter disease-free and overall survival^35–37^.

Although organ preservation is of great importance for patients from a psychological, social, and functional point of view, our study shows that hypopharyngeal carcinomas with high SUV_max_ are at greater risk of treatment failure and should be considered for upfront surgery followed by adjuvant radio(chemo)therapy. With the intensification of nonoperative treatment approaches, an increasing number of patients are experiencing long-term swallowing impairments and functional deficiencies^38^. These late toxic effects are more common following primary chemoradiation in comparison to upfront surgery with postoperative radiotherapy^38,39^. Tschiesner *et al*.^40^ analyzed the functional outcome in patients with advanced head and neck cancer comparing upfront surgery with primary chemoradiation in a cross-sectional, multi-institutional study. They reported no significant difference between the two groups regarding most aspects of functional outcome. However, body functions, including swallowing and weight maintenance, as well as activities and participations, including social relationships, employment, and economic self-sufficiency, were observed to be more problematic in patients receiving initial chemoradiation. Moreover, Jang *et al*.^41^ compared oncological and functional outcomes between initial surgical versus non-surgical treatments for hypopharyngeal cancer in 332 patients. Their data revealed similar oncological outcome and showed even better verbal communication outcomes in advanced-stage hypopharyngeal cancer patients receiving initial chemoradiation. Nevertheless, more patients treated with primary chemoradiation required multiple surgical interventions in the process. This may be explained through a reported failure rate of organ preservation of approximately 46% and also because a significant proportion of survivors with a preserved organ required tracheostomy due to a dysfunctional larynx^41,42^.

Our study goal was to assess tumor response to chemoradiation according to SUV_max_ of the primary tumor but not to assess how the larynx reacted to chemoradiation. Our study might therefore overestimate actuarial organ preservation, as patients who formally achieved organ preservation were assessed as such, regardless of the function of their larynx after chemoradiation. Another limitation of our study is its retrospective design. Furthermore, PET scans were acquired on different scanners, albeit the SUV_max_ is a standardized measure. In addition, the number of patients was relatively low in our study. Differences among groups were, however, sizeable enough to be detected and to avoid beta error^43^. Lastly, owing to the small size of the study population, a sub-analysis depending on HPV status was not performed. In contrast to oropharyngeal cancer, the percentage of HPV-positive tumors of the hypopharynx or larynx is considerably lower and therefore less confounding. Moreover, there is no evidence that HPV-positive hypopharyngeal or laryngeal cancers have a comparable better prognosis as it is the case for oropharyngeal tumors.

In conclusion, SUV_max_ of primary tumor could be used as a predictor of laryngeal preservation before chemoradiation in hypopharyngeal cancer. This information may be of great impact in patient counseling, as SUV_max_ was inversely correlated with the chance of larynx preservation. SUV_max_, however, does not seem to be predictive of outcome in laryngeal cancer.
